# Afatinib Overcomes Pemetrexed-Acquired Resistance in Non-Small Cell Lung Cancer Cells Harboring an EML4-ALK Rearrangement

**DOI:** 10.3390/cells8121538

**Published:** 2019-11-28

**Authors:** Ji-Hyun Kwon, Kui-Jin Kim, Ji Hea Sung, Koung Jin Suh, Ji Yun Lee, Ji-Won Kim, Se Hyun Kim, Jeong-Ok Lee, Jin Won Kim, Yu Jung Kim, Keun-Wook Lee, Jee Hyun Kim, Soo-Mee Bang, Soyeon Kim, Sung-Soo Yoon, Jong Seok Lee

**Affiliations:** 1 Translational Medicine, Department of Medicine, Graduate School, Seoul National University College of Medicine, Seoul 03080, Korea; marioncrepe@chungbuk.ac.kr; 2 Department of Internal Medicine, Chungbuk National University College of Medicine, Cheongju 28644, Korea; 3 Biomedical Research Institute, Seoul National University Bundang Hospital, Seongnam 13620, Korea; kjkim@snubh.org; 4 Department of Internal Medicine, Seoul National University Bundang Hospital, Seoul National University College of Medicine, Seongnam 13620, Korea; R0907@snubh.org (J.H.S.); skjmd0919@snubh.org (K.J.S.); maimatin83@snubh.org (J.Y.L.); jiwonkim@snubh.org (J.-W.K.); sehyunkim@snubh.org (S.H.K.); deafkeller@snubh.org (J.-O.L.); jwkim@snubh.org (J.W.K.); cong1005@snubh.org (Y.J.K.); hmodoctor@snubh.org (K.-W.L.); jhkimmd@snubh.org (J.H.K.); 65368@snubh.org (S.-M.B.); 5 Cancer Research Institute, Seoul National University College of Medicine, Seoul 03080, Korea; thdus81@snu.ac.kr; 6 Department of Internal Medicine, Seoul National University College of Medicine, Seoul 03080, Korea; ssysmc@snu.ac.kr

**Keywords:** pemetrexed, drug resistance, afatinib, lung cancer, EML4-ALK rearrangement

## Abstract

Background: The aim of this study is to elucidate the mechanisms of acquired resistance to pemetrexed in echinoderm microtubule-associated protein-like 4 (EML4)-anaplastic lymphoma kinase (ALK) rearranged non-small cell lung cancer. Methods: We analyzed the sensitivity to pemetrexed and the expression patterns of various proteins after pemetrexed treatment in the cell lines, A549, NCI-H460, NCI-H2228 harboring EML4-ALK variant 3, and NCI-H3122 harboring EML4-ALK variant 1. Pemetrexed-resistant cell lines were also generated through long-term exposure to pemetrexed. Results: The EML4-ALK variant 1 rearranged NCI-H3122 was found to be more sensitive than the other cell lines. Cell cycle analysis after pemetrexed treatment showed that the fraction of cells in the S phase increased in A549, NCI-H460, and NCI-H2228, whereas the fraction in the apoptotic sub-G_1_ phase increased in NCI-H3122. The pemetrexed-resistant NCI-H3122 cell line showed increased expression of EGFR and HER2 compared to the parent cell line, whereas A549 and NCI-H460 did not show this change. The pan-HER inhibitor afatinib inhibited this alternative signaling pathway, resulting in a superior cytotoxic effect in pemetrexed-resistant NCI-H3122 cell lines compared to that in the parental cells line. Conclusion: The activation of EGFR-HER2 contributes to the acquisition of resistance to pemetrexed in EML4-ALK rearranged non-small cell lung cancer. However, the inhibition of this alternative survival signaling pathway with RNAi against EGFR-HER2 and with afatinib overcomes this resistance.

## 1. Introduction

Lung cancer is one of the most common causes of death worldwide, but recent progress in lung cancer treatment has improved its prognosis. In recent years, the use of a targeted agent has been recommended as a frontline treatment depending on the specific mutation of the cancer cells [1,2]. However, genetic mutations that can be targeted by drugs are yet to be found in more than half of lung cancer patients. In the absence of target mutations or during the failure of targeted therapies, combination chemotherapy, including platinum, is generally selected as the primary treatment. Among them, the pemetrexed (PEM)-containing regimen is the most popular therapy, especially in adenocarcinoma patients.

PEM is a multi-targeted antifolate agent that is mainly used for malignant mesothelioma and non-small cell lung cancer (NSCLC). PEM strongly inhibits thymidylate synthase (TS), thereby interfering with folate-dependent nucleic acid synthesis and inhibiting cell division and proliferation, ultimately exhibiting antitumor effects [3,4]. The factors that predict the therapeutic response of PEM are yet to be fully understood. It is known that cancer cells with lower TS are more sensitive to PEM [5,6,7]. However, because PEM has various targets in addition to TS, it affects various enzymes and cell cycle regulators. Therefore, it is presumed that there are various mechanisms promoting or inhibiting the action of PEM.

Investigations have been performed to determine whether genetic mutations targeted by drugs affect the response of PEM. Some researchers have reported that the fusion of the echinoderm microtubule-associated protein-like 4 gene (EML4) with the anaplastic lymphoma kinase (ALK) gene and the reactivity to PEM are related. EML4-ALK rearrangement is found in approximately 5%–10% of NSCLC patients, especially in non-smokers and adenocarcinoma patients [8]. According to Lee et al., NSCLC patients with an EML4-ALK rearrangement showed significantly higher response rates to PEM and improved progression-free survival than control patients [9]. Several studies have also shown that PEM treatment in NSCLC with an EML4-ALK rearrangement is superior in response rate and survival than without this rearrangement [10,11,12,13]. These results suggest that an alteration in the intracellular signaling pathway involved in EML4-ALK has a synergistic effect on the cytotoxic effect of PEM; however, the detailed mechanism has yet to be elucidated.

We identified the change in the response mechanism according to the genetic characteristics of cancer cells by observing changes in the intracellular signaling system after PEM treatment using various NSCLC cell lines, including cell lines with an EML4-ALK rearrangement. In addition, we established PEM-resistant cell lines to investigate the mechanism of resistance to PEM and to discover a process to overcome this resistance.

## 2. Materials and Methods

### 2.1. Materials

Abemaciclib, adriamycin, afatinib, cisplatin, crizotinib, fluorouracil (5-FU), gefitinib, lapatinib, methotrexate (MTX), pemetrexed, and suberoylanilide hydroxamic acid (SAHA) were purchased from Selleckchem (Houston, TX, USA). The following antibodies against the following proteins were purchased from Santa Cruz Biotechnology (Dallas, TX, USA): poly (ADP-ribose) polymerase (PARP, sc-8007), Glyceraldehyde 3-phosphate dehydrogenase (GAPDH, sc-47724), and β-actin (sc-130656). Antibodies against protein kinase B (AKT, cs#4685), phospho-AKT S473 (cs#4058), phospho-AKT T308 (cs#9275), cleaved caspase-9 (cs#7237), cyclin B (cs#4138), epidermal growth factor receptor (EGFR, cs#2646), p-EGFR (cs#2236), erb-b2 receptor tyrosine kinase 2 (ERBB2/HER2, cs#2165), phospho-ERBB2 (cs#2243), ERBB3/HER3 (cs#4754), p-ERBB3/HER3 (cs#2842), mitogen-activated protein kinase kinase (MEK, cs#13033), phospho-MEK S217/221 (cs#9154), phospho-MEK S298 (cs#98195), extracellular-signal-regulated kinase 1/2 (ERK1/2, cs#9102), p-ERK1/2 (cs#4377s), and thymidylate synthase (TS, cs#5449) were purchased from Cell Signaling Technology (Danvers, MA, USA). Mitomycin C (MMC), propidium iodide (PI), RNAse, isopropanol, and sodium dodecyl sulfate (SDS) were purchased from Sigma Aldrich (St Louis, MO, USA). Phosphatase buffer saline (PBS) and fetal bovine serum (FBS) were purchased from Gibco (Grand Island, NY, USA). Roswell Park Memorial Institute (RPMI) 1640 and Dulbecco’s modified Eagle’s medium (DMEM) were purchased from Welgene (Daejeon, Korea). Human recombinant fibroblast growth factor (rhFGF) and rhEGF proteins were purchased from R&D Systems (Minneapolis, MN, USA). FITC-annexin V was purchased from BD Biosciences (San Diego, CA, USA). All chemicals and reagents used were of analytical grade and were obtained from commercial sources.

### 2.2. Cell Culture

The human A549 and NCI-H460 cells were purchased from the Korean Cell Line Bank (KCLB, Seoul, Korea), and NCI-H2228 and NCI-H3122 cells were obtained from the American Type Culture Collection (ATCC, VA, USA). A549, NCI-H460, NCI-H2228, and NCI-H3122 cells were maintained in RPMI 1640 with 10% FBS, 4 mM L-glutamine, and 1% P/S at 37 °C with 5% CO_2_. The most recent authentication of each cell line was performed using AmpFLSTR Identifiler PCR Amplification Kit (Applied Biosystems, Foster, CA, USA) by the KCLB on 12 November 2019 (Supplementary Table S1). A 3530xL DNA Analyzer (Applied Biosystems) and a GeneMapper v5 (Applied Biosystems) were used for DNA fingerprinting analysis.

### 2.3. Cell Proliferation Assay

A cell proliferation assay was performed with the CellTiter-Glo luminescent cell viability assay (Promega, Madison, WI, USA) according to the manufacturer’s instructions. On day 0, 96-well plates were seeded with 3000 cells/well and incubated overnight. The next day (day 1), cells were treated with the appropriate compounds. On day 4, the plates were incubated for 1 h at room temperature and 100 μL of CellTiter-Glo reagent was added to each well, followed by mixing on an orbital shaker for 5 min. Luminescence was measured on a GloMax 96-well luminometer from Promega (Madison, WI, USA).

### 2.4. Cell Cycle Analysis

Cells were seeded in 100-mm plates and grown overnight, then subjected to the appropriate drug treatment for 24 h. After trypsinization, the cells were washed twice in PBS, fixed overnight at 4 °C in ethanol, washed three times in PBS, and incubated in PBS containing 20 μg/mL PI and 100 μg/ml RNAse at 37 °C for 30 min. After washing in PBS, cells were resuspended in 1 mL PBS and examined using a FACSCalibur flow cytometer (BD Biosciences, Franklin Lakes, NJ, USA). Cell cycle distribution was determined using FlowJo software (Tree Star, Ashland, OR, USA).

### 2.5. Western Blot Analysis

Cell lysates were clarified by centrifugation at 12,000× *g* for 30 min at 4 °C. Protein concentration in the supernatant was measured by the Bradford assay (BioLegend, San Diego, CA, USA). Proteins (20 μg) were separated by SDS polyacrylamide gel electrophoresis, transferred to a polyvinylidene difluoride membrane (Bio-Rad, Hercules, CA, USA) blocked in blocking buffer containing 5% skim milk, and then probed overnight with primary antibodies. Secondary antibodies conjugated with horseradish peroxidase (1:4000 dilution; Bio-Rad) were applied for 1 h. Immunoreactivity was detected by enhanced chemiluminescence (Biosesang, Seongnam, Korea) and a ChemiDoc Touch imager (Bio-Rad).

### 2.6. Colony Forming Assay

Cells were seeded in 6-well plates and grown for 72 h before being subjected to the appropriate treatment for 10 days. A medium change occurred at regular time intervals. After 10 days of culture at 37 °C with 5% CO_2_, colonies were washed with PBS and stained with Coomassie Brilliant Blue for 30 min at room temperature, then washed with water and air-dried. The colonies were photographed using the ChemiDoc Touch (Bio-Rad) and measured using ImageJ software (National Institutes of Health, Bethesda, MD, USA).

### 2.7. Receptor Tyrosine Kinase Protein Array

Human RTK phosphorylation antibody array C1 kit (AAH-PRTK-1-8) and human EGFR phosphorylation array C1 kit (AAH-PER-1-4) were obtained from RayBiotech (Norcross, GA, USA). The assay for the RTK array was conducted according to the manufacturer’s instructions. Lung cancer cell lysates prepared from NCI-H3122 R cells were diluted and incubated with the array’s membranes. The density of the immunoreactive area obtained on the RTK arrays was then analyzed by Chemidoc touch (Bio-Rad). 

### 2.8. Quantitative Reverse Transcriptase Polymerase Chain Reaction (qRT-PCR)

Total RNA was isolated from lung cancer cells using TRIzol reagent (Invitrogen Life Technologies, Grand Island, NY, USA), following the manufacturer’s instructions. RNA concentrations and purity were estimated by determining the A260/A280 ratio with a Nanodrop2000 spectrophotometer (Invitrogen). The complementary DNA (cDNA) were synthesized by cDNA Synthesis Kit (iNtRON Biotechnology, Daegu, Korea) according to the manufacturer’s instructions. qRT-PCR was carried out using SYBR Green in a Thermal Cycler DiceTM Real Time System 3 (DAKARA Bio Inc). The sequences of the oligonucleotide primer were: amphiregulin (AREG) sense (5′-ATA GAG CAC CTG GAA GCA GTA ACA-3;) and antisense (5′-TGT GAG GAT CAC AGC AGA CAT AAA G-3′); betacellulin (BTC) sense (5′-CTT CAC TGT GTG GTG GCA GAT G-3′) and antisense (5′-ATG CAG TAA TGC TTG TAT TGC TTG G -3′); epidermal growth factor (EGF) sense (5′-GGA CAA CAG TGC TTT GTA AAT TGT G-3;) and antisense (5′-CCA GTG TGA CTG TCT GCT TTA ACC-3′); EGFR sense (5′- TTG CCA AGG CAC GAG TAA CAA G-3;) and antisense (5′-ACT GTG TTG AGG GCA ATG AGG AC-3′); HER2 sense (5′-CTG ATG GGT TAA TGA GCA AAC TGA-3′) and antisense (5′-CCA AAT TCT GTG CTG GAG GTA GAG-3′); HER3 sense (5′- GGG AGC ATT TAA TGG CAG CTA-3′) and antisense (5′-GAA TGG AAT TGT CTG GGA CTG G-3′); epiregulin (EREG) sense (5′-GCT CTC AGC TGA TGT GTC CTG TA-3′) and antisense (5′-AAC TGG GTT ATT ATG TGG CCT TG-3′); heparin-binding EGF-like growth factor (HB-EGF) sense (5′-GGG CAT GAC TAA TTC CCA CTG A-3′) and antisense (5′-GCC CAA TCC TAG ACG GCA AC-3′); transforming growth factor alpha (TGF-α) sense (5′-TGG CCG GGA TGG ACT AAT G-3′) and antisense (5′-CTT CTG TGA CTG GGC AGG TTG-3′); and 18s sense (5′-GCT TAA TTT GAC TCA ACA CGG GA-3′) and antisense (5′- AGC TAT CAA TCT GTC AAT CCT GTC-3′). The expression levels were calculated using the 2^ΔΔCt^ method after correcting for differences in PCR efficiencies. Values were expressed relative to those of the control group.

### 2.9. RNA Interference

Cells were transfected with control, ALK siRNA, EGFR siRNA, or HER2 siRNA (Bioneer, Daejeon, Republic of Korea; SN-1003, SN-238-1, SN-1956-1, or SN-2064-1, respectively) at a concentration of 100 nM using Lipofectamine 2000 reagent (Life Technologies, Carlsbad, CA, USA). After 24 h, the cells were harvested for analysis of knockdown efficiency and then were used for further analysis. 

### 2.10. Statistical Analysis

Student’s *t*-test was used to compare differences. Differences among multiple groups were evaluated by one-way analysis of variance (ANOVA) followed by Duncan’s multiple range test using SPSS 23.0 software (IBM, Armonk, NY, USA). Mean values with different letters (for example, a, b, c, and d) indicate statistically significant differences (*p* < 0.05). In contrast, values having the same letters indicate no significant differences (*p* > 0.05).

## 3. Results

### 3.1. Effect of Pemetrexed on Cell Viability, Cell Cycle, and Apoptosis-Associated Protein Expression In Lung Cancer Cell Lines

**Figure 1:**
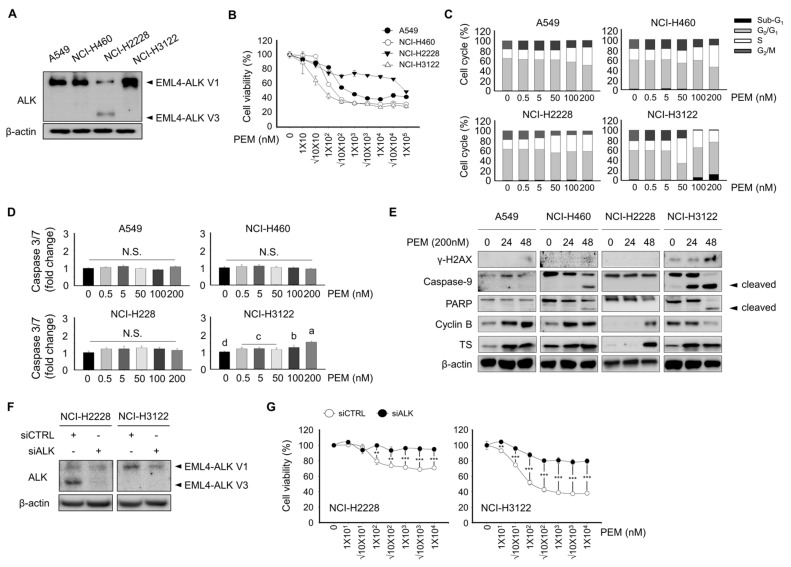
Effect of pemetrexed on cell viability, cell cycle, and apoptosis in lung cancer cell lines. (**A**) A549, NCI-H460, NCI-H2228 EML4-ALK V3, and NCI-H3122 EML4-ALK V1 cells were evaluated for their basal expression of ALK protein by Western blotting; β-actin served as the loading control. (**B**) Cell viability assay. A549, NCI-H460, NCI-H2228, and NCI-H3122 cells were treated with the indicated concentrations of PEM for 3 days. (**C**) Cell cycle analysis by PI staining and flow cytometry. A total of 1 × 10^6^ cells were seeded in 60-mm plates and treated with 0, 0.5, 5, 10, 50, 100, and 200 nM of PEM for 24 h. Data are presented as histograms (black, Sub-G_1_; gray, G_0_/G_1_ phase; white, S phase, and dark gray, G_2_/M phase). (**D**) Caspase 3/7 activity was quantified 24 h after PEM treatment in A549, NCI-H460, NCI-H2228, and NCI-H3122 cells. Means with different letters (a, b, c, and d) indicate statistically significant differences (*p* < 0.05). (**E**) γ–H2AX, Caspase-9, PARP, cyclin B, and TS expression in A549, NCI-H460, NCI-H2228, and NCI-H3122 cells, as determined by Western blotting; β-actin served as the loading control. (**F**) Expression of ALK was evaluated using Western blotting in NCI-H2228 and NCI-H3122 with or without siALK; β-Actin served as the loading control. (**G**) Cell viability assay. NCI-H2228 and NCI-H3122 cells with or without siALK were treated with the indicated concentrations of PEM for 3 days.

Several clinical studies have reported the potential benefits of PEM-based chemotherapy on EML4-ALK rearrangement NSCLC [14,15,16]. Although favorable clinical outcomes have been achieved with PEM treatment in EML4-ALK rearrangement NSCLC, it is still necessary to address the underlying mechanism of PEM in the EML4-ALK rearrangement NSCLC. To investigate the correlation between an EML4-ALK rearrangement and PEM sensitivity, we first evaluated the protein expression of ALK in four lung cancer cell lines by Western blotting. As shown in Figure 1A, ALK showed weak expression levels in the A549 and NCI-H460 cell lines. In contrast, NCI-H2228 or NCI-H3122 cells, which harbor the EML4-ALK variant (V) 3 or EML4-ALK V1 rearrangement, showed a prominent protein expression of ALK. Subsequently, we determined the effect of A549, NCI-H460, NCI-H2228, and NCI-H3122 cell viability following PEM treatment (Figure 1B). The half maximal inhibitory concentration (IC_50_) values for PEM were 0.328 ± 0.024, 0.137 ± 0.008, 199.697 ± 12.522, and 0.077 ± 0.015 nM in A549, NCI-H460, NCI-H2228, and NCI-H3122, respectively. Such findings indicated that NCI-H3122 cells harboring EML4-ALK V1 exhibit a greater sensitivity to PEM than the A549, NCI-H460, and NCI-H2228 EML4-ALK V3 cell lines. Further, we showed that upon ALK knockdown with RNAi, PEM was not able to decrease cell viability in siALK NCI-H2228 and siALK NCI-H3122 cells compared to control cells (Figure 1F,G), indicating that the EML4-ALK rearrangement characteristics contribute to the sensitivity to PEM.

Flow cytometry analysis was performed to evaluate the effect of PEM on cell cycle transition in lung cancer cell lines (Figure 1C). PEM inhibited the progression at the S phase checkpoint to the G_2_ phase in A549, NCI-H460, and NCI-H2228 harboring EML4-ALK V3 cells. Notably, the fraction of sub-G_1_ strikingly increased (*p* < 0.05), suggesting elevated apoptotic cells by PEM in NCI-H3122 cells harboring the EML4-ALK V1 rearrangement. 

The effect of PEM on apoptosis was examined using a caspase 3/7 activity assay. PEM (200 μM) was insufficient to cause apoptosis in A549, NCI-H460, and NCI-H2228 cells (Figure 1D). However, in NCI-H3122, caspase 3/7 activity significantly increased after PEM treatment. These results were confirmed by Western blot, which revealed a strong induction of γ-H2AX, cleaved-caspase 9, and cleaved-PARP proteins after PEM treatment in a dose-dependent manner in NCI-H3122 cells compared to A549, NCI-H460, and NCI-H2228 cells. In A549, NCI-H460, and NCI-H2228 cells, PEM increased the protein expression of cyclin B, whereas a decreased expression of cyclin B by PEM was observed in NCI-H3122 cells. PEM also induced the protein expression of TS irrespective of EML4-ALK status among the lung cancer cell lines. 

### 3.2. Development of Pemetrexed-Resistant Lung Cancer Cell Lines

**Figure 2:**
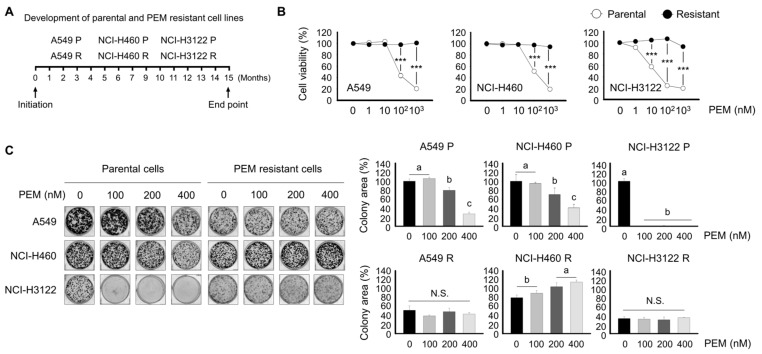
Development of parental and PEM-acquired resistant cell lines and evaluation of PEM sensitivity in A549, NCI-H460, and NCI-H3122 cells. (**A**) A549, NCI-H460, and NCI-H3122 cells were treated with or without 200 nM of PEM for 15 months. (**B**) Cell viability assays. A549 P, A549 R, NCI-H460 P, NCI-H460 R, NCI-H3122 P, and NCI-H3122 R cells were treated with the indicated concentrations of PEM for 3 days (***, *p* < 0.001 versus corresponding control). (**C**) Colony formation assays were conducted in A549 P, A549 R, NCI-H460 P, NCI-H460 R, NCI-H3122 P, and NCI-H3122 R cell lines. Means with different letters (a, b, and c) indicate statistically significant differences (*p* < 0.05; N.S., not significant).

Acquired PEM-resistant cell lines were generated by exposing A549, NCI-H460, or NCI-H3122 cells harboring the EML4-ALK rearrangement treated with or without 200 nM of PEM for a 15-month period (Figure 2A). After 15 months of continued exposure to 0 or 200 nM of PEM, cells became parent or resistant and were named A549 P, A549 R, NCI-H460 P, NCI-H460 R, NCI-H3122 P, and NCI-H3122 R. Cells were then cultured in the presence of 0 to 10^3^ nM PEM to evaluate their PEM-acquired resistance (Figure 2B). PEM-resistant cell lines showed approximately 60% less sensitivity to PEM compared to their corresponding parental cells. 

To investigate PEM resistance features, we evaluated colony formation and the cell migration ability of A549 R, NCI-H460 R, and NCI-H3122 R cells and their parental cells. Consistent with Figure 2B, PEM treatment resulted in a significantly reduced colony area in NCI-H3122 P, and lower colony density in NCI-H3122 P cells than in A549 P and NCI-H460 P cells (Figure 2C). 

Notably, A549 R and NCI-H460 R cells had reduced colony-forming capacity compared to their parental cells. NCI-H3122 R also had a very low to nearly absent colony-forming capacity. 

### 3.3. Alternative Activation of the Receptor Tyrosine Kinase (RTK) Family as a Major Mechanism Mediating PEM-Acquired Resistance

**Figure 3:**
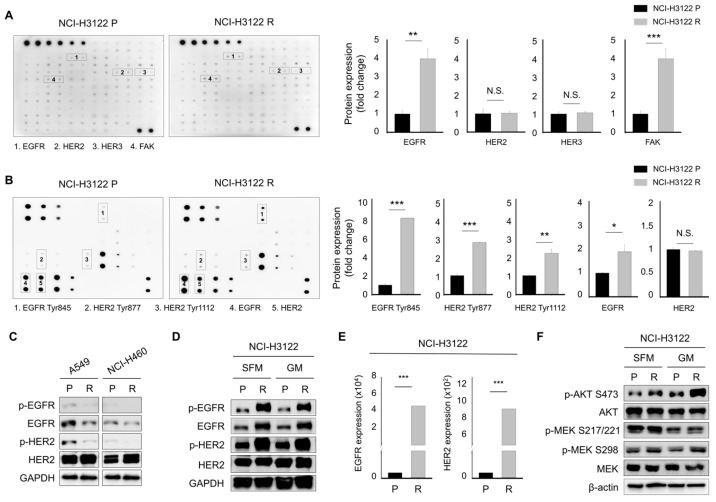
The PEM-acquired resistant NSCLC cell harboring EML4-ALK rearrangement model displays overexpression of the RTK signaling pathway. (**A**,**B**) RTK and EGFR phosphorylation arrays that were altered in NCI-H3122 R compared to the parental cell line. The densitometric ratio of duplicate spots for activated RTK and EGFR proteins to the internal loading controls on the RTK phosphorylation and EGFR phosphorylation array was calculated using Image J software (**, *p* < 0.01; ***, *p* < 0.001; N.S. not significant). (**C**,**D**) Western blotting analysis of A549 P, A549 R, NCI-H460 P, NCI-H460 R, NCI-H3122 P, and NCI-H3122 R cells for phospho-EGFR, EGFR, phospho-HER2, and HER2 expression was performed. GAPDH served as the loading control (SEM, serum-free media; GM; growth media). (**E**) EGFR and HER2 mRNA expression were determined in NCI-H3122 P and NCI-H3122 R cells by qRT-PCR. Fold change in EGFR, HER2, and HER3 mRNA in NCI-H3122 R cells was compared to that in parental cells (***, *p* < 0.001; N.S. not significant). (**F**) Western blotting analysis of NCI-H3122 P and NCI-H3122 R cells for p-AKT S473, AKT, p-MEK S217/221, p-MEK S298, and MEK. β-Actin served as the loading control (SEM, serum-free media; GM; growth media).

We evaluated the molecular basis of the PEM-resistance effect. Several studies have suggested that the receptor tyrosine kinase (RTK) family, including EGFR, HER2, HER3, and HER4, is one of the major drug resistance mechanisms against pharmacological therapeutic reagents in different cancers, including NSCLC [17,18,19]. To investigate whether the activation of RTK occurs in NCI-H3122 P and NCI-H3122 R cells, we employed human RTK and EGFR phosphorylation arrays and observed that the NCI-H3122 R cell line displayed a significant increase in phospho-EGFR, EGFR, and phospho-HER2 expression compared to NCI-H3122 P (Figure 3A,B). An increase in the expression level of phospho-FAK, which is one of the downstream targets of EGFR and HER2 proteins, was also found in NCI-H3122 R cells.

Consistent with Figure 3A,B, Western blotting analysis revealed that an obvious increase in the total level of EGFR protein was detected in NCI-H3122 R cells, with increased expression levels of phospho-EGFR and phospho-HER2, whereas no significant changes were observed in A549 R or NCI-H460 R cells (Figure 3C,D). However, the expression levels of total HER3 and phospho-HER3 proteins were not altered in NCI-H3122 R cells compared to NCI-H3122 P cells (data not shown). We examined the expression levels of the mRNA of these RTKs by qRT-PCR. As a result, we found that the expression levels of EGFR and HER2 mRNA were significantly increased in NCI-H3122 R cells compared to NCI-H3122 P (Figure 3E). The RTK family regulates the expression levels of AKT and MEK proteins [20], and the expression levels of phospho-AKT S473, phospho-MEK S217/S2221, and phospho-MEK S298 protein were relatively increased in NCI-H3122 R cells compared to NCIH3122 P cells (Figure 3F).

### 3.4. Effect of Acquired PEM Resistance on the Receptor Tyrosine Kinase (RTK) Ligands in NCI-H3122 Cells

We proceeded to determine how EGFR and HER2 are activated. Previous studies have suggested that the ligands of RTK secreted through the autocrine and paracrine signaling in a tumor microenvironment are the major contributors of RTK activation [21,22,23]. We, therefore, examined the expression levels of EGFR ligands including AREG, BTC, EGF, EREG, HB-EGF, and TGF-α in NCI-H3122 R cells (Figure S1A–F). The analysis of qRT-PCR revealed that the expression levels of AREG, BTC, EGF, EREG, HB-EGF, and TGF-α were not changed in NCI-H3122 R cells compared to NCI-H3122 P cells, indicating that the expression of EGFR and HER2 is potentially activated by a cellular genetic alteration in NCI-H3122 R cells. 

### 3.5. Overcoming PEM Resistance by Using RNAi and Tyrosine Kinase Inhibitor (TKI)

The aforementioned evidence that NCI-H3122 R cells showed constitutive expression of EGFR and HER2 suggest that the EGFR and HER2 targeting inhibition may overcome PEM-acquired resistance. 

**Figure 4:**
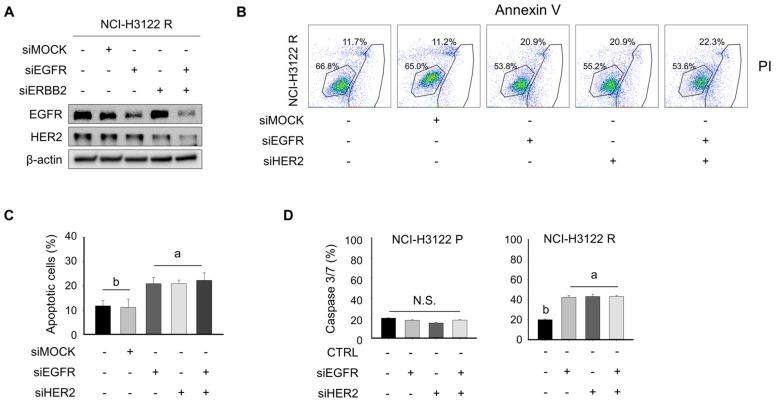
Knockdown of EGFR-HER2 exerts a pro-apoptotic effect in NCI-H3122 R cells. (**A**) NCI-H3122 R cells were transfected with siEGFR and/or siHER2 and were incubated for 24 h. Cells were harvested and the expression of EGFR and HER2 was evaluated with Western blotting. β-Actin served as the loading control. (**B**) Apoptosis was evaluated using flow cytometry of Annexin V-PI double-stained NCI-H3122 R cells after transfection with siEGFR and/or siHER2 for 24 h. The Y-axis represents the PI-labeled population, whereas the X-axis represents the Annexin V positive cells. The left lower gating (Annexin V-, PI-) indicates normal cells, whereas the right lower gating (Annexin V+, PI-) and the right upper gating (Annexin V+, PI+) are the early and late apoptotic cells, respectively. (**C**) Data are presented as histograms. Means with different letters (a and b) indicate statistically significant differences (*p* < 0.05; N.S., not significant). (**D**) Caspase 3/7 activity was quantified 24 h after transfection with siEGFR and/or siHER2 in NCI-H3122 R cells. Means with different letters (a and b) indicate statistically significant differences (*p* < 0.05; N.S., not significant).

Therefore, we first knocked down EGFR and HER2 using RNA interference in NCI-H3122 R cells and determined the transfection efficacy, a portion of apoptosis, and caspase 3/7 activity. As shown in Figure 4A, siEGFR and/or siHER2 decreased the protein expression of EGFR and/or HER2 in NCI-H3122 R cells. In addition, single EGFR or HER2 knockdown significantly increased a portion of apoptosis and caspase 3/7 activity (Figure 4B–D). In contrast, the synergistic cell death of NCI-H3122 R cells transfected with both siEGFR and siHER2 was not observed. 

**Figure 5:**
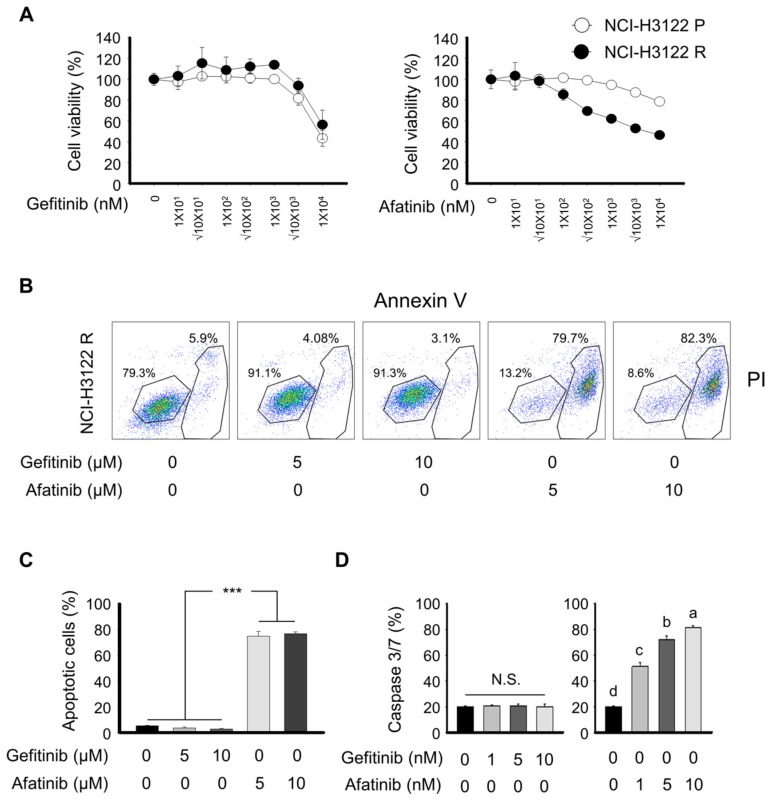
RTK inhibition sensitizes the acquired PEM-resistant model of NSCLC. (**A**) NCI-H3122 P and NCI-H3122 R cells were treated with the indicated concentrations of PEM for 3 days. (**B**) Apoptosis was evaluated by flow cytometry of Annexin V-PI double-stained NCI-H3122 R cells after treatment with gefitinib or afatinib for 24 h. The Y-axis represents the PI-labeled population, whereas the X-axis represents the Annexin V positive cells. The left lower gating (Annexin V-, PI-) indicates normal cells, whereas the right lower gating (Annexin V+, PI−) and the right upper gating (Annexin V+, PI+) are the early and late apoptotic cells, respectively. (**C**) Data are presented as histograms (*** *p* < 0.001). (**D**) Caspase 3/7 activity was quantified 24 h after gefitinib or afatinib treatment in NCI-H3122 R cells. Means with different letters (a, b, c, and d) indicate statistically significant differences (*p* < 0.05; N.S., not significant).

We next treated NCI-H3122 P and NCI-H3122 R cell lines with EGFR tyrosine kinase inhibitor (TKI) and analyzed cell viability, apoptosis, and caspase 3/7 activity. To increase the robustness of our observation, we used different EGFR TKIs such as gefitinib and afatinib. In the presence of gefitinib, cell viability and apoptotic cells were not different between the NCI-H3122 P and NCI-H3122 R cell lines (Figure 5A,B). Afatinib noticeably enhanced cellular sensitivity to NCI-H3122 R compared to NCI-H3122 P cells, as demonstrated by a sustained shift in IC_50_ value with treatment above the 100 nM afatinib concentrations. Notably, a significant increase in the incidence of apoptosis occurred with 5 and 10 μM of afatinib in NCI-H3122 R cells (Figure 5C). Concentrations of 5 and 10 μM gefitinib resulted in an insufficient number of apoptotic cells. 

To confirm our findings, we performed the analysis of caspase 3/7 activity on NCI-H3122 P and NCI-H3122 R in the presence of gefitinib or afatinib (Figure 5D). Consistent with the results of cell viability and flow cytometry, increased caspase 3/7 activity was observed with afatinib in NCI-H3122 R cells. In addition, we tested other molecular inhibitors and cytotoxic agents, including lapainib, cisplatin, methyltransferase (MTX), adriamycin, abemaciclib, SAHA, crizotinib, and fluorouracil (5-FU), on whether these pharmaceutical regents could overcome PEM-acquired resistance in NCI-H3122 cells (Supplementary Figure S2). These drugs have no statistically significant difference between NCI-H3122 P and NCI-H3122 R based on the IC_50_ values.

### 3.6. Effect of the Tyrosine Kinase Inhibitor (TKI) on RTK Downstream Cascade and Colony Formation in NCI-H3122 R Cells

**Figure 6:**
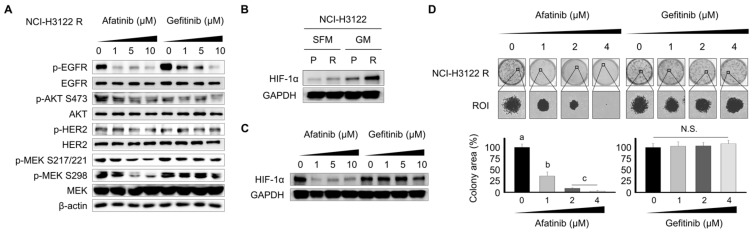
RTK inhibition abrogates RTK expression in NCI-H3122 R cells. (**A**) NCI-H3122 R cells were treated with afatinib or gefitinib. The expression levels of phospho-EGFR, EGFR, phospho-AKT S473, AKT, phospho-HER2, HER2, phospho-MEK S217/221, phospho-MEK S298, and MEK were assessed by Western blotting in NCI-H3122 R cells. β-Actin served as the loading control. (**B**) Basal expression of HIF-1α was evaluated with Western blotting. GAPDH served as the loading control (SEM, serum-free media; GM; growth media). (**C**) NCI-H3122 R cells were treated with afatinib of gefitinib. The expression levels of HIF-1α were evaluated with Western blotting in NCI-H3122 R cells. GAPDH served as the loading control. (**D**) Colony-forming assays were performed in NCI-H3122 R cells. Means with different letters (a, b, and c) indicate statistically significant differences (*p* < 0.05; N.S., not significant).

To elucidate the underlying mechanisms involved in the overcoming of TKI-mediated PEM acquired resistance, the expression levels of the RTK signaling pathway were analyzed by Western blot (Figure 6A). The TKIs, afatinib and gefitinib, decreased the expression levels of phospho-EGFR and phospho-AKT S473. Furthermore, afatinib suppressed the expression levels of phospho-HER2 and phospho-MEK protein in NCI-H3122 R cells. However, gefitinib was found to be insufficient for suppressing the expression levels of phospho-HER2 and phospho-MEK protein. Hypoxia-inducible factor 1 alpha (HIF-1α) has been recognized as a gefitinib resistance molecule in NSCLC cells [24]. Interestingly, we found that the basal expression levels of the HIF-1α protein were higher in NCI-H3122 R cells that acquired resistance to PEM (Figure 6B). These data indicated that the aberrant overexpression of HIF-1α might play a partial role in PEM-acquired resistance in NSCLC cells harboring an EML4-ALK rearrangement. As shown in Figure 6C, afatinib dramatically attenuated the expression level of HIF-1α in NCI-H3122 cells. In addition, colony-forming assays were performed to functionally investigate the inhibitory capacity of afatinib and gefitinib in NCI-H3122 R cells. Afatinib at 1, 2, and 4 μM significantly inhibited the colony growth of NCI-H3122 R cells (Figure 6D) compared to control, but gefitinib had an ineffective potency against NCI-H3122 R cells. 

Together, our data suggest that PEM-acquired resistance is strongly associated with the constitutive expression of both EGFR and HER2 protein owing to the potential genetic alteration, and the inhibition of those receptor activities may confer the PEM-acquired resistance in NSCLC cells harboring an EML4-ALK rearrangement. 

## 4. Discussion

PEM is one of the most important cytotoxic agents used in non-squamous cell carcinoma of the lung. The main target of PEM is known as TS, but the mechanism of PEM reactivity and resistance cannot be fully explained by TS alone. It is difficult to predict the response of PEM to TS expression in tissues of real patients, and the depth and duration of responses to PEM in the same type of lung cancer vary. Several researchers have reported that NSCLC harboring an EML4-ALK rearrangement is more sensitive to PEM than those with wild type ALK, but the mechanism has not been studied in depth. The ALK receptor is a classical receptor tyrosine kinase. EML4-ALK rearrangement leads to activation of downstream signaling and contributes to cell survival and proliferation by interacting with the PI3K/AKT, JAK/STAT, and RAS/RAF/MEK/ERK [25,26]. The difference in PEM sensitivity based on the presence of ALK rearrangement suggests that PEM may have some inhibitory effects on these signaling pathways.

We found that EML4-ALK-rearranged cell lines decreased their viability more rapidly during PEM treatment than wild type ALK cell lines. We also found that the superior sensitivity to PEM of EML4-ALK-rearranged cells was accompanied by early onset of apoptosis during PEM treatment. Because the main mechanism of PEM is to inhibit nucleic acid synthesis, PEM-alone treatment is generally known to induce S-phase arrest [27]. On the other hand, ALK inhibitors, including crizotinib, increase the sub-G_1_ apoptotic population in cells with the EML4-ALK rearrangement [28]. In our experiments, PEM increased the sub-G_1_ population, similar to crizotinib, in EML4-ALK-rearranged cells, whereas S-phase arrest occurred in PEM-treated wild-type ALK cells. Despite harboring the EML4-ALK rearrangement, the NCI-H2228 cell line had different response patterns to PEM than NCI-H3122. This is thought to result from the difference between the variant type of ALK rearrangement of NCI-H3122 and NCI-H2228. Previous studies have reported that the NCI-H2228 cell line with EML4-ALK V3 rearrangement is less sensitive to ALK inhibitors than cells with EML4-ALK V1 [29]. This finding supports the notion that PEM induces apoptosis by affecting the ALK-associated signaling pathway, especially those specific to the EML4-ALK V1 rearrangement and consequently exhibits an additional anti-tumor response in the EML4-ALK-rearranged cell lines. Considering the fact that PEM treatment induces the same cell cycle changes as an ALK inhibitor, and that the pattern of sensitivity according to the variant type of EML4-ALK rearrangement is the same as that of an ALK inhibitor, it can be assumed that PEM works in a way similar to an ALK inhibitor in cells with the EML4-ALK rearrangement.

In addition to the different reaction mechanisms for PEM, EML4-ALK-rearranged cells have unique resistance mechanisms compared to ALK-negative cells. In the present study, we showed that the activation of EGFR and HER family signaling accompanied by acquired PEM resistance was restricted in EML4-ALK-rearranged cells. This finding suggested that the major mechanism responsible for cell survival and proliferation is the conversion from ALK association to the EGFR-HER family. It has been known that activation of the HER family-related pathway in ALK-positive cells contributes to the acquisition of resistance to ALK inhibitors. Tanizaki et al. reported that blocking the ALK signaling pathway using the ALK inhibitor in EML4-ALK-rearranged lung cancer cells converts cell survival from the ALK pathway-dependent mechanism to the HER family [19]. Another investigator reported the involvement of the insulin-like growth factor-1 receptor (IGF1R) and HER3 in the resistance of the next-generation ALK inhibitor, alectinib [30]. These reports have suggested a hypothesis in which the increase in the levels of proteins associated with the EGFR or HER family in cells exposed to ALK inhibitors is another causative mechanism for obtaining resistance to ALK inhibitors.

In our study, EML4-ALK-rearranged cells manifested an increase in HER2 activation in the presence of PEM, similar to the results of ALK inhibitors in the previous studies. This finding suggests that PEM activates the HER pathway similar to ALK inhibitors, which leads to the development of resistance to PEM. Consistent with our results, Wang et al. reported that the efficacy of PEM-containing chemotherapy was most pronounced in NSCLC harboring ALK rearrangement or ROS1 mutations, whereas NSCLC with HER mutations was poorly responsive to PEM treatment [31]. Given these results, it can be assumed that activation of the HER signal pathway may play a role in acquiring PEM resistance. In addition, PEM-resistant and parental cell lines showed no difference in responsiveness to crizotinib. We, therefore, assumed that PEM has an inhibitory effect on ALK signaling, but the target of PEM is a component of the downstream signaling pathway, and not of ALK itself.

Given that the development of resistance to PEM in ALK-positive cells is associated with the activation of the alternative signaling system, including the HER family, this leads to the possibility that EGFR-HER inhibition will have a therapeutic effect on lung cancer that has acquired PEM resistance. Previous studies have shown that intracellular changes by PEM are associated with responsiveness to EGFR-HER inhibitors. Li et al. reported the use of the EGFR inhibitor, erlotinib, after PEM was deemed effective in ALK wild type NSCLC cells [32]. They reported that PEM increases the phosphorylation of EGFR and AKT, and blocking PEM-activated EGFR-dependent PIK3-AKT pathway with erlotinib after PEM treatment may provide additional cytotoxicity. This finding was observed regardless of EGFR and KRAS mutations. Although conducted using NSCLC cells without ALK rearrangement, this study provides a clue to the fact that intracellular changes by PEM are related to the EGFR signaling system and the utility of a treatment strategy using EGFR inhibitors after PEM. 

In our experiments, the pan-HER inhibitor afatinib had no effect on EML4-ALK-rearranged parental cells, but its cytotoxic activity increased after obtaining PEM resistance. The EGFR inhibitor gefitinib was ineffective because various HER molecules, in addition to EGFR, may contribute to PEM reactivity and resistance in ALK-positive cells. These findings suggest that the inhibition of several HER molecules is required to overcome the survival inhibitory effect by PEM in EML4-ALK-rearranged cells.

We also showed that the acquisition of PEM resistance was accompanied by overexpression of HIF-1α protein. HIF-1α is a factor that is activated in a hypoxic state to regulate cell survival and apoptosis, and is known to mainly have an anti-apoptotic function [33]. Another reason for not responding to gefitinib despite the increased expression of EGFR in PEM-resistant cell lines may be the activation of HIF-1α by PEM. Our study showed that afatinib, on the other hand, attenuate the increased levels of HIF-1α in PEM-resistant cell lines. Therefore, we suggest that the pan-HER inhibitor may be a more appropriated therapeutic option for EML4-ALK-rearranged NSCLC after prolonged exposure to PEM, even though it has not been initially identified as having HER overexpression. Further experiments are needed in the future for the development of a clinically useful treatment strategy.

## 5. Conclusions

This study investigated the previously unknown mechanism of responsiveness and resistance to PEM in EML4-ALK-rearranged lung cancer. The development of PEM resistance in lung cancer with an ALK rearrangement is associated with the activation of the EGFR-HER family, which provides a rationale for the use of the pan-her inhibitor, afatinib, to overcome PEM-acquired resistance. Further study is required to achieve the clinical relevance of these findings.

## Supplementary Materials

The following are available online at https://www.mdpi.com/2073-4409/8/12/1538/s1. Supplementary Table S1. DNA fingerprinting of lung cancer cell lines. Supplementary Figure S1. Effects of acquired PEM resistance on RTK ligand mRNA expression in NCI-H3122 P and NCI-H3122 R cells. Supplementary Figure S2. Effects of molecular inhibitors and cytotoxic agents on the acquisition of PEM resistance in NCI-H3122 cells.
