# Supplementary material for: Afatinib Overcomes Pemetrexed-Acquired Resistance in Non-Small Cell Lung Cancer Cells Harboring an EML4-ALK Rearrangement
Source: Cells. 2019 Nov 28;8(12):1538. doi: 10.3390/cells8121538 (PMC6953071; doi:10.3390/cells8121538)
Supplement: Supplementary file 1 [file cells-08-01538-s001.pdf]

cells

MDPI

Supplementary Information

Supplementary Table S1. The DNA fingerprinting of lung cancer cell lines.

	D8S1179	D21S11	D7S820	CSF1PO	D3S1358	TH01	D13S317	D16S539	D2S1338	D19S433	Vwa	TPOX	D18S51	Amelogenin	D5S818	FGA
A549	13,14	29	8,11	10,12	16	8,9.3	11	11,12	24	13	14	8,11	14,17	X,Y	11	23
A549P	13,14	29	8,11	10,12	16	8,9.3	11	11,12	24	13	14	8,11	14,17	X,Y	11	23
A549R	13,14	29	8,11	10,12	16	8	11	11,12	24	13	14	8,11	14,17	X,Y	11	23
NCI-H460	12	30	9	11,12	15,18	9.3	13	9	17,25	14	17	8	13,15	X,Y	9,10	21,23
NCI-H460P	12	30	9,12	11,12	15,18	9.3	13	9	17,25	14	17	8	13,15	X,Y	9,10	21,23
NCI-H460R	12	30	9,12	11,12	15,18	9.3	13	9	17,25	14	17,18	8	13,16	X,Y	9,10	21,23
NCI-H3122P	13,15	28,29	8,12	11,12	16	7,9.3	10,12	11,12	17,23	14	16	10	13,16	X,Y	11,12	18,21
NCI-H3122R	13,15	28,29	8,12	11,12	16	7,9.3	10,12	11,12	17,23	14	16	10	13,16	X,Y	11,12	18,21
NCI-H2228	13	28,30	11	12	19,18	7,8	11	11,13	18	14,15	15,17	11	16	x	12	21,22

A549P_STR results 2019.10.31

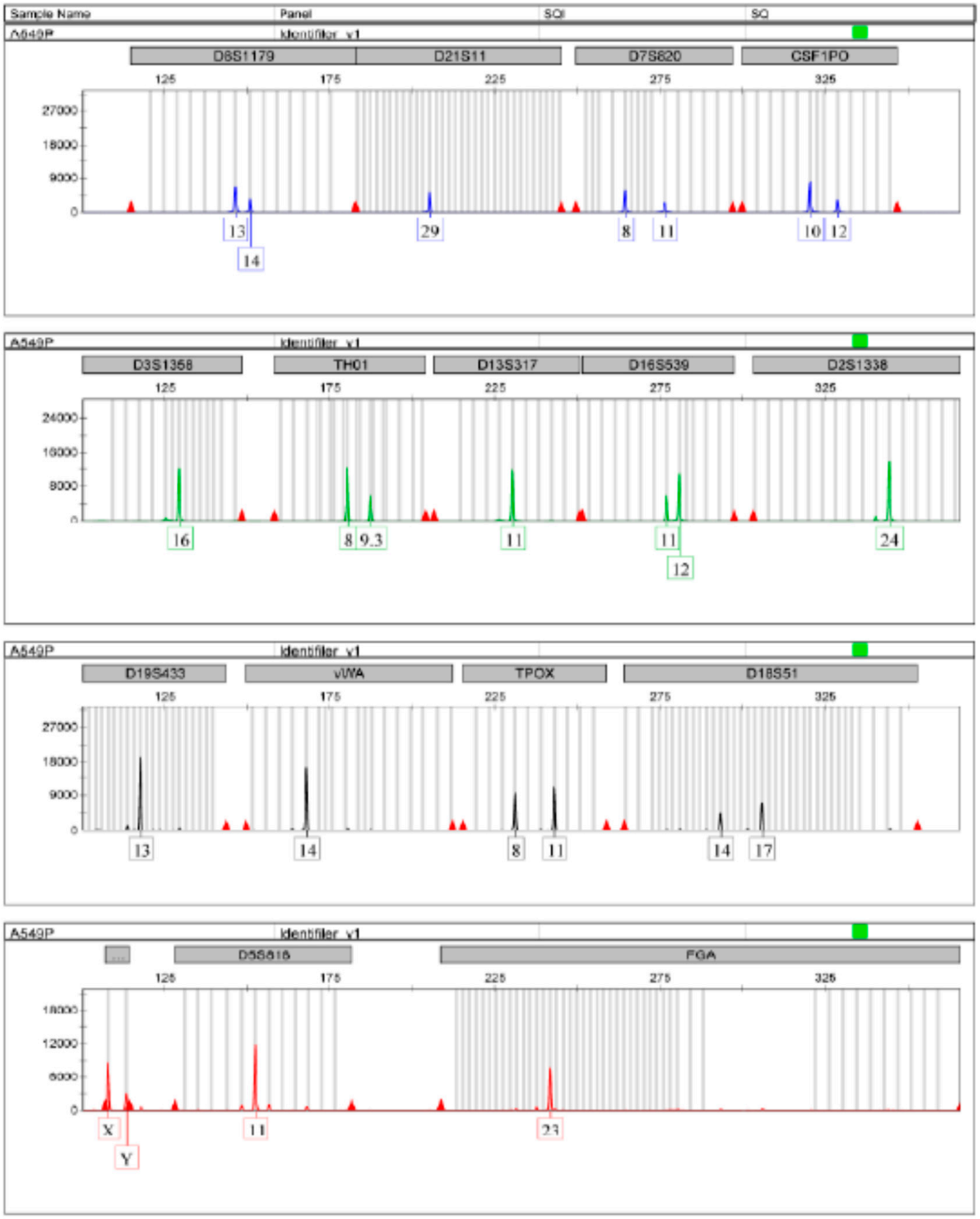

A549R_STR results 2019.10.31

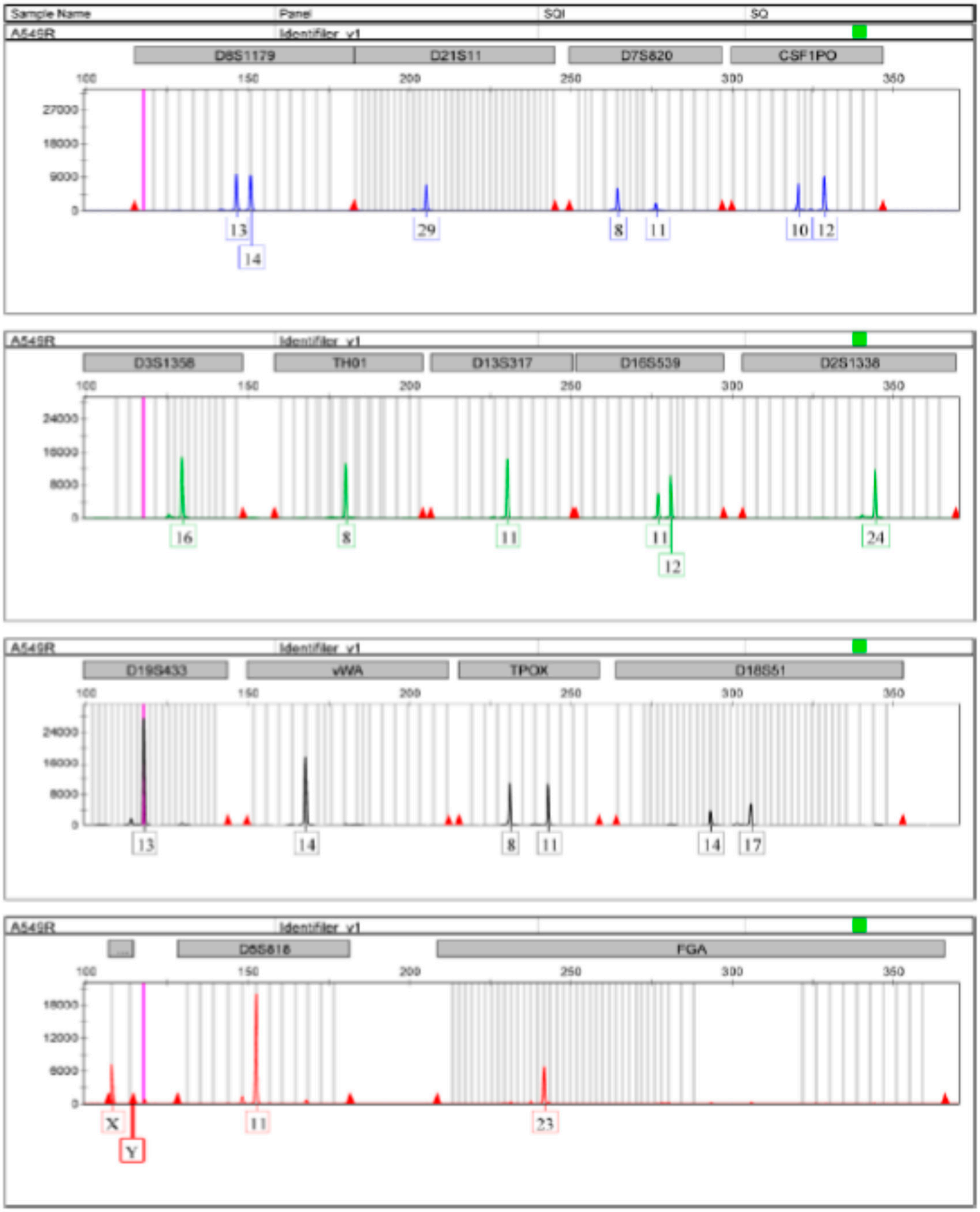

NCI-H460P_STR results 2019.10.31

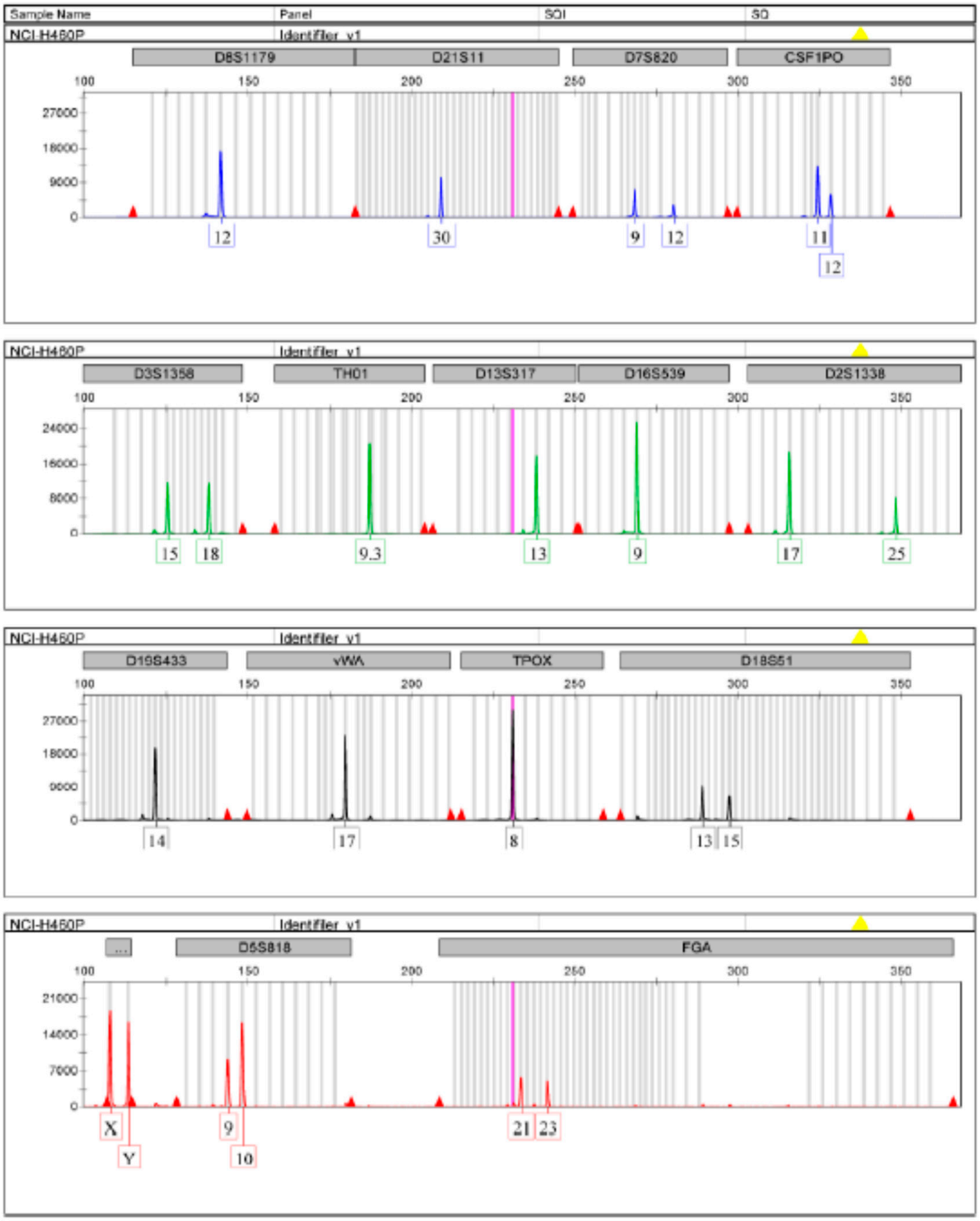

NCI-H460R _STR results 2019.10.31

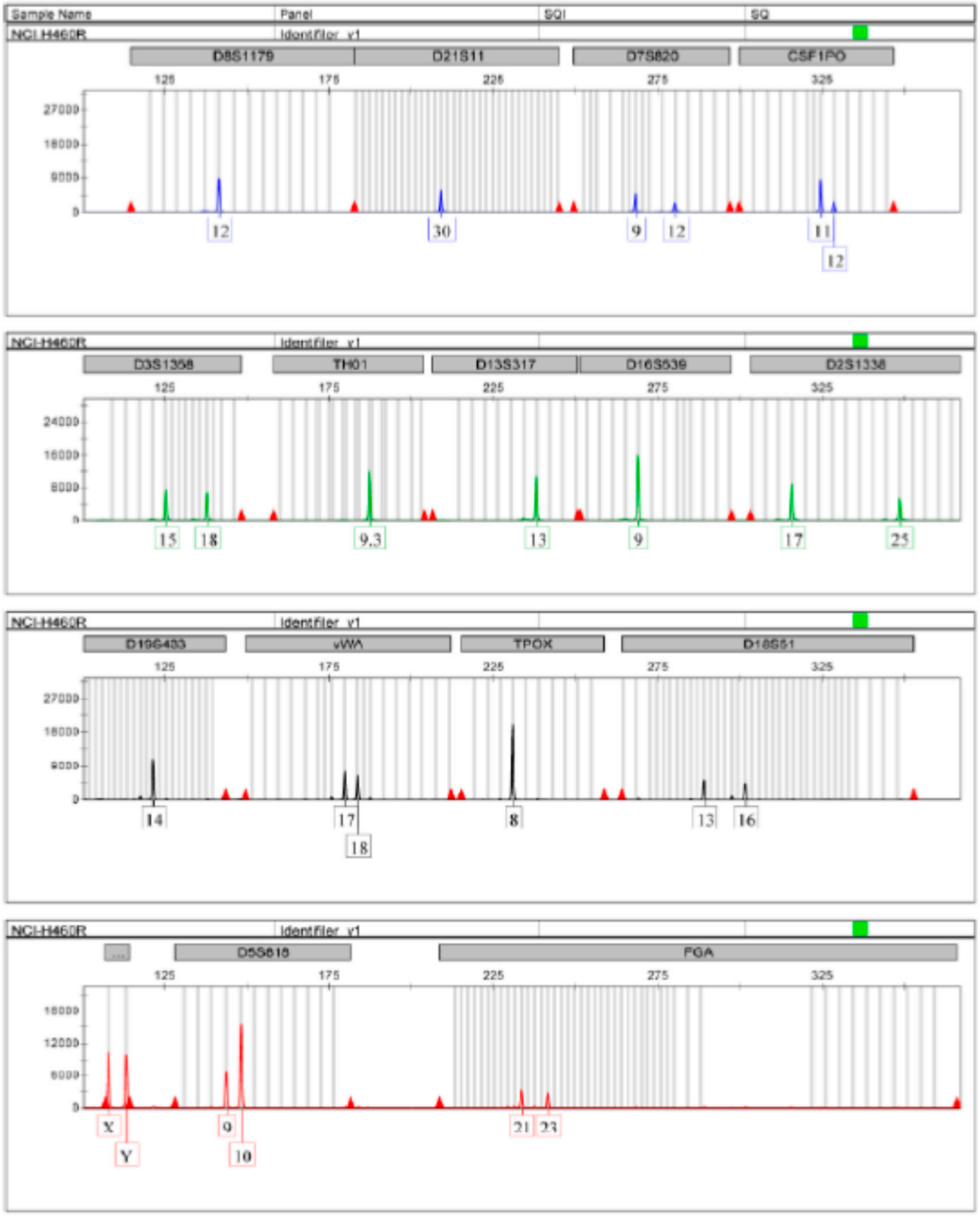

NCI-H3122P_STR results 2019.10.31

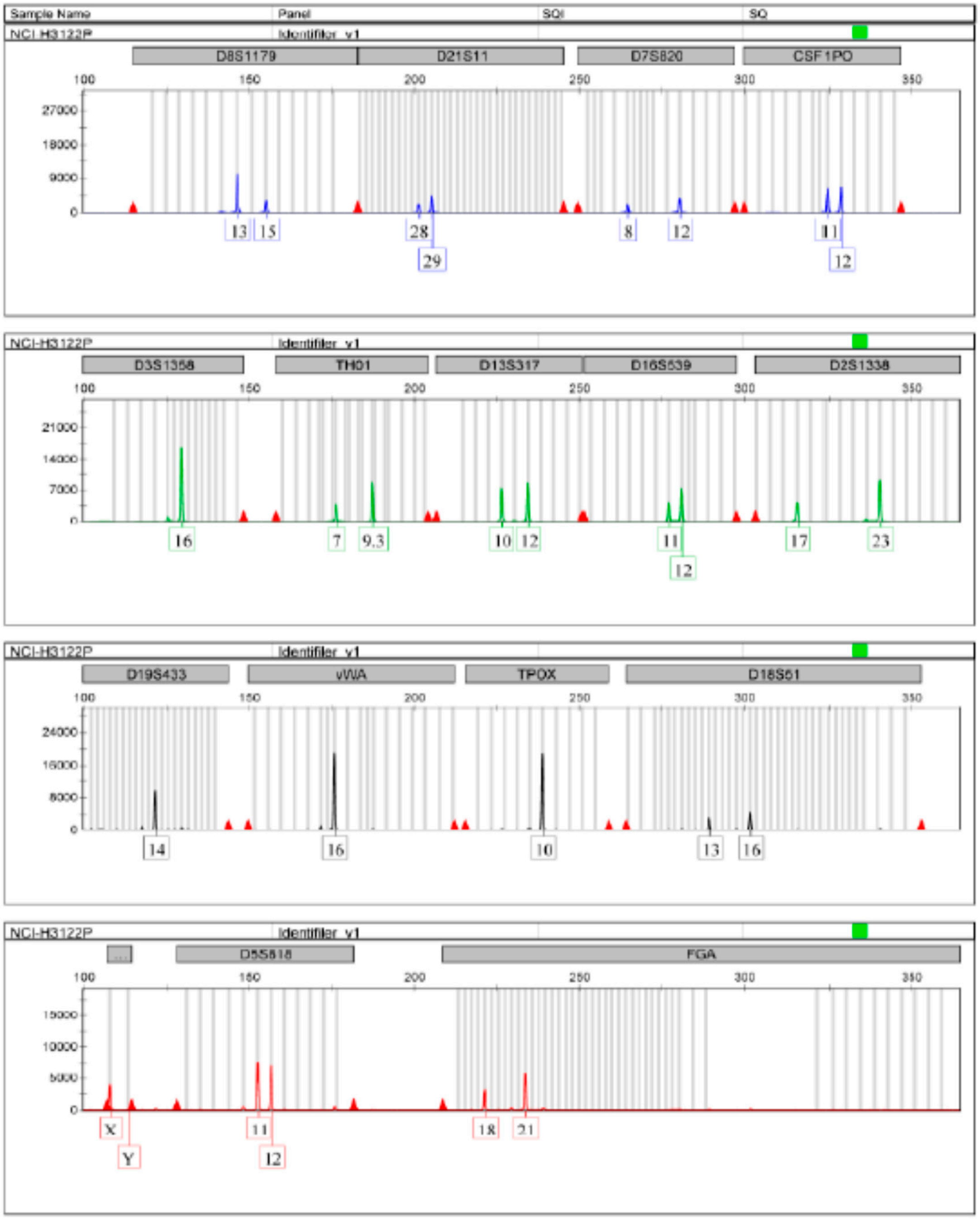

NCI-H3122R_STR results 2019.10.31

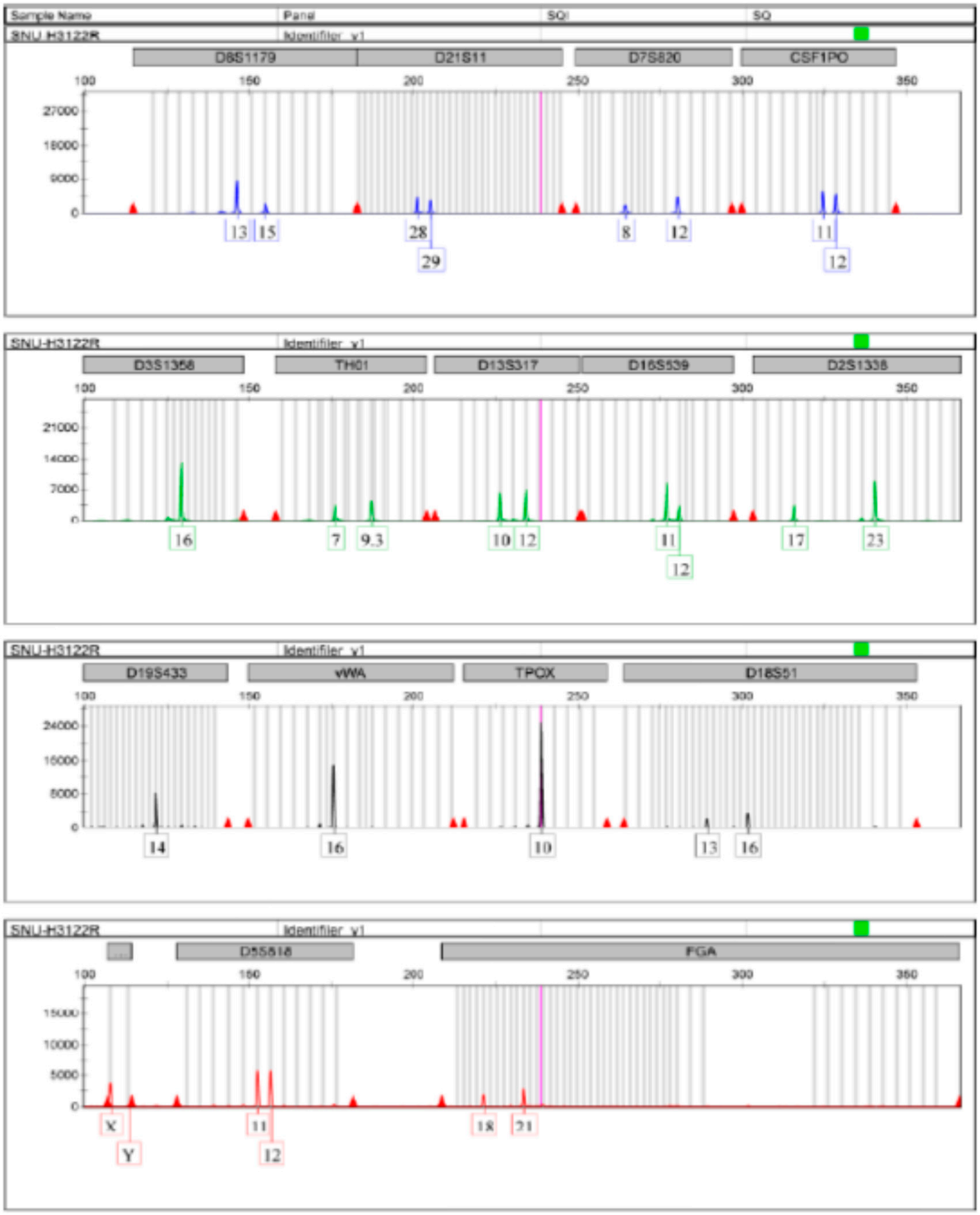

NCI-H2228_STR results 2019.11.12

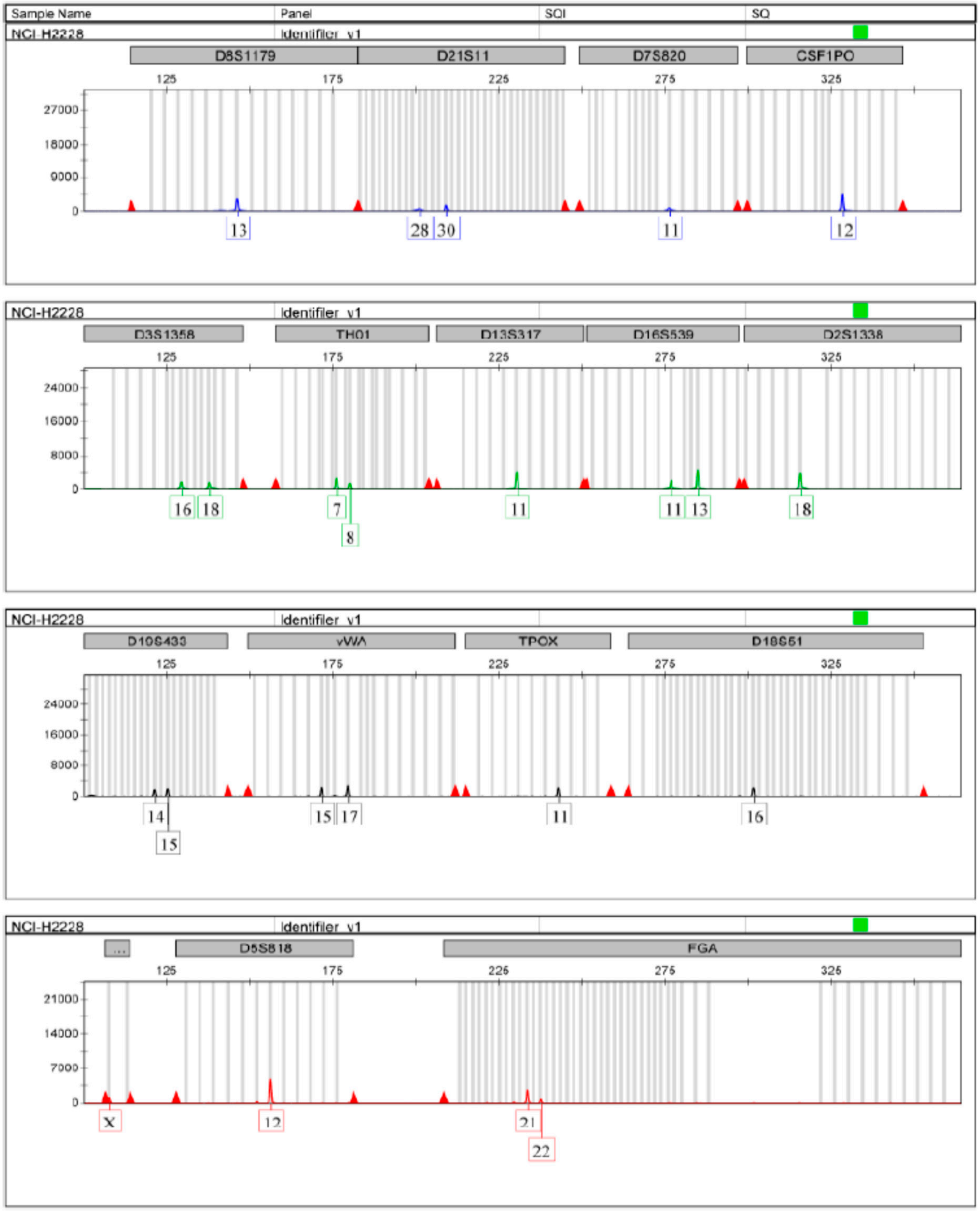

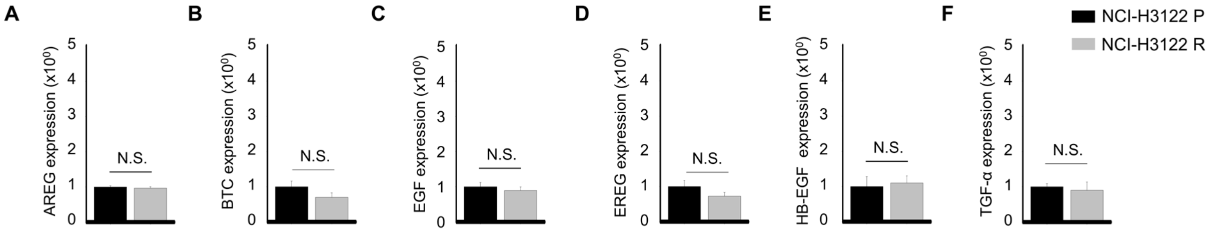

Supplementary Figure S1. Effect of PEM-acquired resistance on RTK ligand mRNA expression in NCI-H3122 P and NCI-H3122 R cells. **A-F.** AREG, TRC, EGF, EREG, HB-EGF, and TGF-α mRNA expression levels were determined in NCI-H3122 P and NCI-H3122 R cells by qRT-PCR. Fold change in AREG, TRC, EGF, EREG, HB-EGF, and TGF-α mRNA in NCI-H3122 R cells was compared to that in parental cells (N.S., not significant).

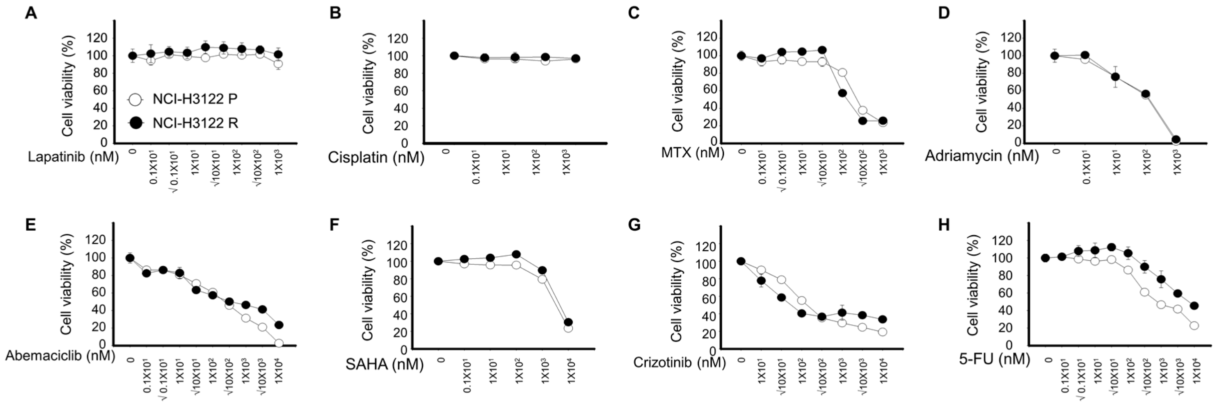

Supplementary Figure S2. Effect of molecular inhibitors and cytotoxic agents on the acquisition of PEM resistance in NCI-H3122 cells. **A-H.** NCI-H3122 P and NCI-H3122 R cells were treated with the indicated concentrations of lapatinib, cisplatin, MTX, Adriamycin, abemaciclib, SAHA, crizotinib, and 5-FU for 3 days.
